# Prognostic significance of incidental suspected transthyretin amyloidosis on routine bone scintigraphy

**DOI:** 10.1007/s12350-020-02396-7

**Published:** 2020-10-22

**Authors:** Olli Suomalainen, Jaagup Pilv, Antti Loimaala, Sorjo Mätzke, Tiina Heliö, Valtteri Uusitalo

**Affiliations:** 1 Cardiology Department, Kymenlaakso Central Hospital, Kotkantie 41, 48210 Kotka, Finland; 2 Clinical Physiology and Nuclear Medicine, Helsinki University Hospital and University of Helsinki, Helsinki, Finland; 3 Heart and Lung Center, Helsinki University Hospital and University of Helsinki, Helsinki, Finland

**Keywords:** Amyloidosis, Transthyretin, Bone scintigraphy

## Abstract

**Background:**

Transthyretin amyloidosis (ATTR) is an occasional incidental finding on bone scintigraphy. We studied its prognostic impact in elderly patients.

**Methods:**

The study population consisted of 2000 patients aged over 70 years who underwent bone scintigraphies with clinical indications in three nuclear medicine departments (Kymenlaakso, Jorvi and Meilahti hospitals) in Finland. All studies were performed using ^99m^Technetium labeled hydroxymethylene diphosphonate (HMDP). ATTR was suspected in patients with ≥grade 2 Perugini grade uptake (grade 0-3). Heart-to-contralateral ratio (H/CL) of ≥ 1.30 was considered positive for ATTR. The overall and cardiovascular mortality were obtained from the Finnish National Statistical Service.

**Results:**

There were a total of 1014 deaths (51%) and 177 cardiovascular deaths (9%) during median follow-up of 4 ± 2 years. ATTR was suspected in 69 patients (3.6%) of which 54 (2.7%) had grade 2 and 15 (.8%) had grade 3 uptake and in 47 patients (2.4%) by H/CL ratio. In multivariate analyses age, bone metastasis, H/CL ratio and grade 3 uptake were independent predictors of overall and cardiovascular mortality. Grade 2 uptake was a predictor of cardiovascular mortality.

**Conclusions:**

A suspected ATTR as an incidental finding on bone scintigraphy predicts elevated overall and cardiovascular mortality in elderly patients.

**Electronic supplementary material:**

The online version of this article (10.1007/s12350-020-02396-7) contains supplementary material, which is available to authorized users.

## Introduction

Amyloidosis is a systemic disease characterized by low-molecular-weight amyloid aggregating into tissues.^1^ Cardiac amyloidosis is primarily caused by transthyretin (ATTR) or light chain amyloid accumulation which lead to progressive heart failure and conduction abnormalities.^1^ Cardiomyopathy due other amyloid subtypes is rare but can occur.^2^,^3^ ATTR may be hereditary or caused by non-mutated transthyretin deposits in elderly.^1^ Recent developments in transthyretin stabilizing therapies underline the importance of its early diagnosis.^4^ Unfortunately, diagnostic delay of ATTR is frequently long by which time the quality of life is already reduced and response to treatment is poor due to advanced cardiomyopathy.^4^,^5^

Scintigraphy is an accurate method for noninvasive diagnosis of ATTR.^1^,^3^ Since both amyloid and bone scintigraphy utilizes the same bone avid radiotracers, ATTR is occasionally an incidental finding on bone imaging. In previous studies, the incidence of cardiac uptake on routine bone scintigraphy has been .4%-2% and mostly seen in elderly patients.^6^,^7^ However, the prognostic significance of incidental ATTR on bone scintigraphy is currently unknown. Scintigraphy detects both subclinical and advanced ATTR cardiomyopathy.^8^ Thus, in elderly individuals symptomatic cardiomyopathy might not manifest itself during their lifetime considering that the primary indications for bone scintigraphy are oncologic. In an autopsy series, up to 25% of individuals aged over 85 years had myocardial transthyretin accumulation, which raises the possibility of medicalization of subclinical ATTR in elderly patients.^9^

In our study, we evaluate the prognostic significance of incidental suspected ATTR on routine bone scintigraphy. We hypothesize that incidental cardiac uptake is associated with both overall and cardiovascular mortality which would demonstrate its clinical significance in an elderly patient population.

## Methods

The study was a retrospective multicenter study consisting of 2000 patients older than 70 years who underwent bone scintigraphy between 2012 and 2018 at Meilahti University Hospital (N = 1045), Jorvi Central Hospital (N = 516) or Kymeenlaakso Central Hospital (N = 439) in Finland. The most common indications for imaging were prostate cancer (N = 1426) and breast cancer (N = 384) whereas other miscellaneous indications were less frequent (N = 190). Exclusion criteria for our study were nondiagnostic image quality for assessment of cardiac uptake and known or suspected amyloidosis at the time of bone scintigraphy. Overall and cardiovascular mortality statistics were obtained from the Finnish National Statistical Service (Tilastokeskus). Overall mortality is defined as death of any cause including cardiovascular deaths. Cardiovascular deaths were identified using the obtained death certificates with a diagnosis code suggesting cardiovascular death as the underlying cause of death (I00-I99) in the International Classification of Diseases (ICD-10). The diagnosis of heart failure at the time of bone scintigraphy was acquired using patient records in patients with suspected ATTR. The local ethics committee approved the study and it was performed according to the declaration of Helsinki.

### Bone Scintigraphy

**Figure 1:**
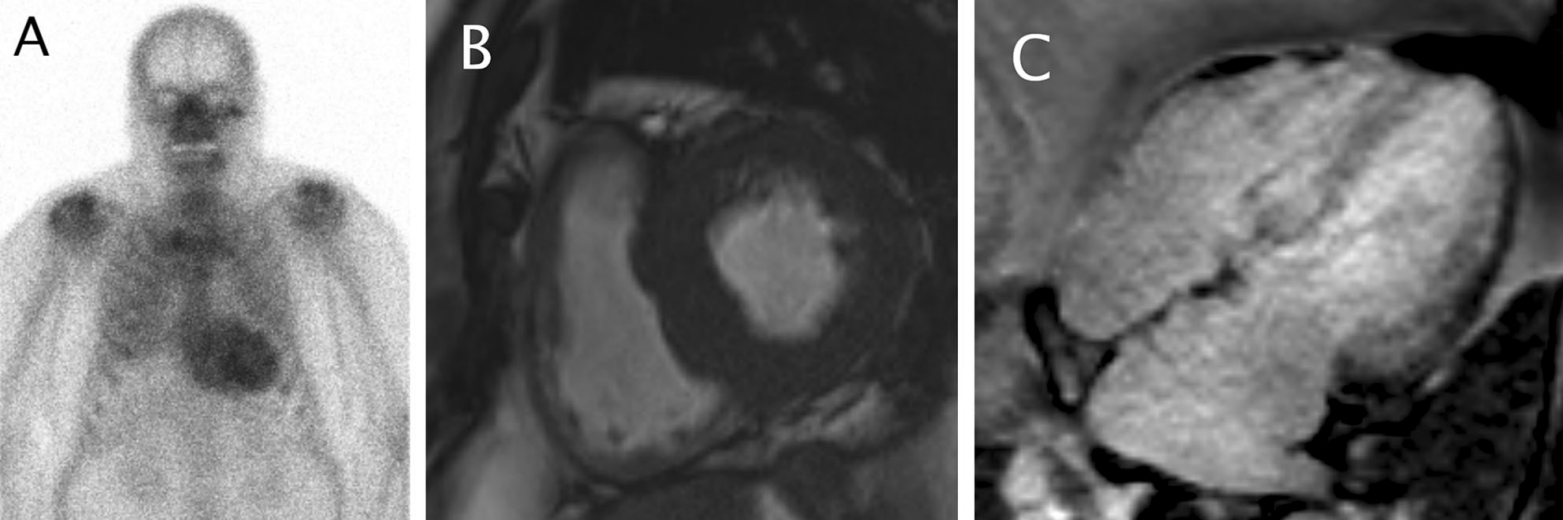
Incidental cardiac and pulmonary uptake on bone scintigraphy imaged to exclude prostate cancer bone metastases. Heart-to-contralateral ratio (H/CL) was 1.83. (**A**) After 5 years the same patient was evaluated in cardiology department for suspected chronic heart failure and left ventricular hypertrophy. Magnetic resonance imaging demonstrated thick septum of 22 mm (**B**) and wide-spread late gadolinium enhancement characteristic of cardiac amyloidosis (**C**). Laboratory testing for light chain disease was negative and diagnosis of ATTR cardiomyopathy was made

All bone scintigrams were imaged using ^99m^technetium labeled hydroxymethylene diphosphonate (HMDP) and anterior and posterior whole-body planar views were obtained using a standard gamma camera at 3 hours after radiotracer injection. Other imaging planes or single-photon emission tomography (SPECT) and fusion images with computed tomography (CT) were viewed as available. Cardiac uptake of all bone scintigrams were graded using the Perugini grade as previously described; no uptake (grade 0), less than bone (grade 1), equal to bone (grade 2) and greater than bone (grade 3).^3^,^10^ Grading of cardiac uptake was done retrospectively for research purposes only. ATTR was suspected in patients with greater than or equal to grade 2 (G2) Perugini grade uptake. Three investigators graded the bone scintigraphy images of our study population for cardiac uptake (OS, JP and VU). All patients with a positive scan (≥G2) were reviewed by one nuclear medicine physician (VU). Heart-to-contralateral ratios (H/CL) were calculated for all patients with any cardiac uptake. A ratio of ≥ 1.30 was considered positive for ATTR.^11^,^12^ Clinical data and other imaging findings were available at the time of bone scintigraphy analysis. The information on the presence of bone metastases was gathered using image reports and when necessary, reviewing of other imaging data available. Images were viewed and analyzed using Impax (Agfa Healthcare, Mortsel, Belgium) and Hermes (Hermes Medical Solutions, Stockholm, Sweden) softwares. Figure 1. demonstrates a patient with incidental cardiac uptake and subsequent cardiac magnetic resonance imaging (MRI) confirming ATTR.

### Statistical Analyses

Continuous variables were described as mean and standard deviation. Categorical variables were described as percentage and frequency. Normally distributed variables were compared using the Student’s T test and non-normally distributed variables with the Mann–Whitney *U* test. The association between cardiac uptake and adverse events were studied using the Kaplan–Meier method. In addition, hazard ratios (HR) and confidence intervals (CI) were calculated using multivariate Cox proportional hazard analyses. Variables with a *P* value < .10 in univariate analyses were selected for further multivariate analysis. Two-tailed *P* values of < .05 were considered statistically significant. All statistical analyses were performed using the MedCalc software 17.1 (MedCalc Software, Mariakerke, Belgium).

## Results

### Patient Characteristics

Mean age of the study population was 78 ± 6 and 31% were females. Mild grade 1 cardiac uptake was seen in 335 (16.8%), grade 2 in 54 (2.7%) and grade 3 in 15 (.8%) individuals, respectively. ATTR was suspected in 69 individuals (3.5%) who had ≥G2 cardiac uptake. Individuals with suspected ATTR were older (81 ± 6 vs 78 ± 6 years, *P* < .0001) and more frequently male (77 vs 6%, *P* < .01) than the individuals whose studies were not suggestive of cardiac ATTR amyloidosis. The indications for bone scintigraphy were similar in the individuals with ≤G1 and > G1 cardiac uptake (*P* = .61). Of individuals with suspected ATTR, a total of 28 (41%) had known diagnosis of heart failure at the time of bone scintigraphy. The incidence of metastatic bone disease was similar between individuals with and without suspected ATTR (32 vs 33%, *P* = .78). Patient characteristics are shown in Table 1.

**Table 1:** Patient characteristics

Variable	All patients (N = 2000)	≤G1 uptake (N = 1931)	≥G2 uptake (N = 69)	*P* value
Age (years)	78 ± 6	78 ± 6	81 ± 6	< .0001
Female	31%	31%	23%	< .01
Bone metastasis	636 (32%)	613 (32%)	23 (33%)	.78
Indication for bone scintigraphy				
Prostate cancer	1426 (71%)	1377 (71%)	49 (71%)	.61*
Breast cancer	384 (19%)	368 (19%)	16 (23%)	
Other	190 (10%)	186 (10%)	4 (6%)	
Perugini grade				
Grade 0	1596 (79.8%)			
Grade 1	335 (16.8%)			
Grade 2	54 (2.7%)			
Grade 3	15 (.8%)			
H/CL ratio (G1 vs ≥G2)		1.06 ± .11	1.35 ± .34	< .0001
Adverse events				
Follow-up (median ± SD)	3.7 ± 2.1 years	3.7 ± 2.1 years	3.0 ± .3 years	.44
Death	1014 (51%)	972 (50%)	42 (61%)	.09
Cardiovascular death	177 (9%)	159 (8%)	18 (35%)	< .0001

Data are presented as mean ± SD or N (%). *Between all groups.

*G1*, grade 1; *G2*, grade 2; *H/CL*, heart-to-contralateral side.

**Figure 2:**
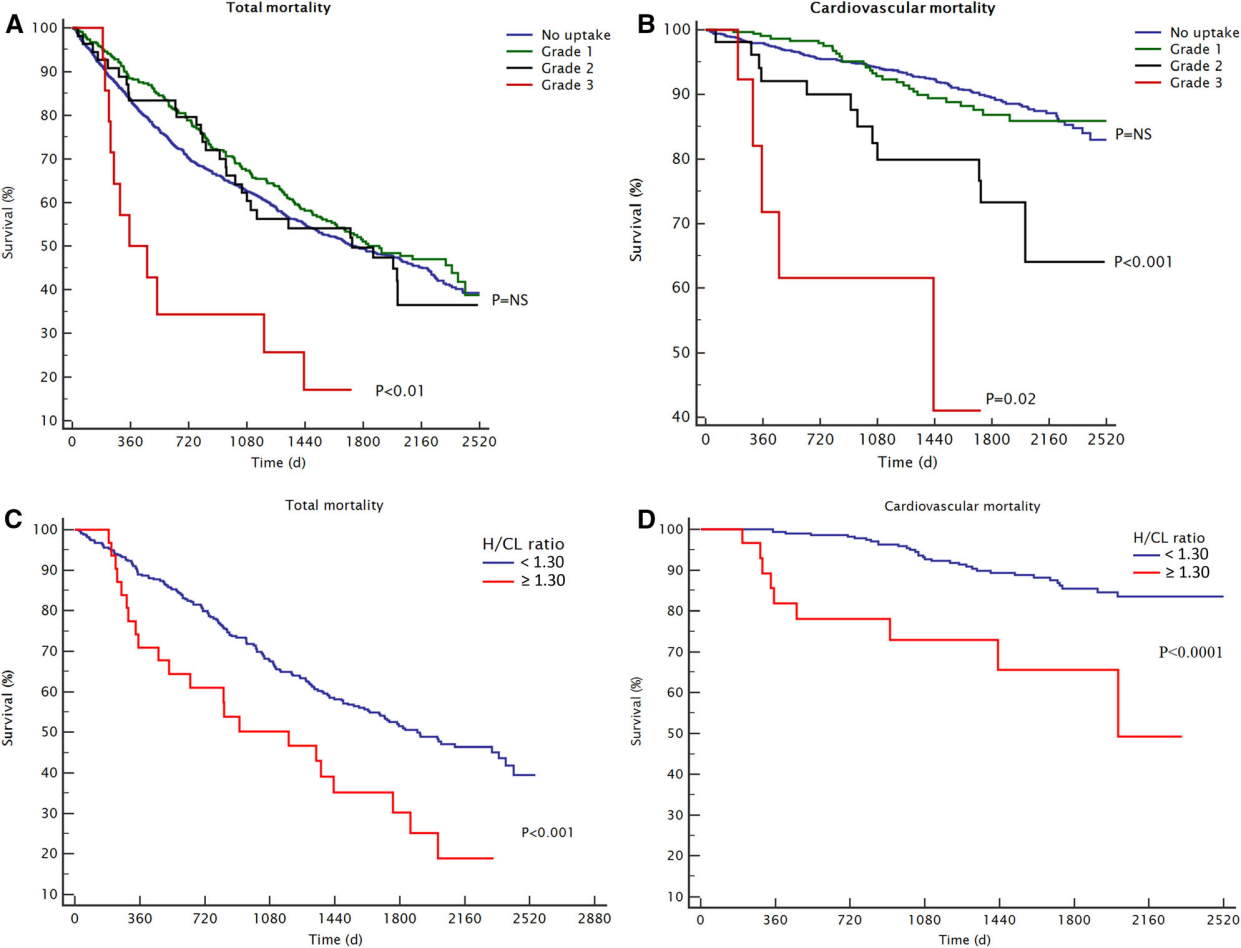
Incidental myocardial uptake on bone scintigraphy and patient prognosis. Overall mortality in patients with different *Perugini grades* of myocardial uptake; no uptake (grade 0), lower than bone (grade 1), equal to bone (grade 2) and higher than bone uptake (grade 3) (**A**). Cardiovascular mortality according to *Perugini grade* (**B**). Overall mortality (**C**) and cardiac mortality (**D**) in patients with suspected transthyretin amyloidosis in quantitative heart-to-contralateral ratio (H/CL) analysis (H/CL ≥ 1.30) compared to patients with lower intensity of cardiac uptake (H/CL < 1.30)

Median follow-up time was 4 ± 2 years. There were a total of 1014 deaths in the study population (51%) of which 177 (9%) were classified as cardiovascular deaths. Figure 2 shows the association between cardiac uptake and patient mortality.

### Suspected ATTR and prognosis

The association between mortality and suspected ATTR is shown in Tables 2 and 3. In univariate analyses age, gender, bone metastasis, grade 3 cardiac uptake and H/CL ratio were predictors of overall mortality (*P* < .05). Age, bone metastasis, grade 3 cardiac uptake were all independent predictors of total mortality in subsequent multivariate analysis (*P* < .05).

**Table 2:** Univariate analysis of patient characteristics and mortality.

Variable	Hazard ratio	Confidence interval	*P* value
Total mortality			
Age	1.07	1.06–1.08	<.0001
Female	.81	.70–.94	<.01
Bone metastasis	4.04	3.56–4. 58	<.0001
Mild cardiac uptake (G1)	.89	.74–1.05	.15
Cardiac uptake (≥G2)	1.28	.94–1.75	.11
Cardiac uptake (G3)	2.80	1.54–5.07	<.001
H/CL ratio	3.93	1.98–7.78	.0001
H/CL ratio ≥ 1.30	1.96	1.25–3.07	<.01
Cardiovascular mortality			
Age	1.13	1.10–1.16	<.0001
Female	.48	.31–.74	<.001
Bone metastasis	2.00	1.43–2.77	<.0001
Mild cardiac uptake (G1)	1.05	.70–1.56	.82
Cardiac uptake (≥G2)	3.41	2.09–5.55	<.0001
Cardiac uptake (G3)	8.70	3.55–21.30	<.0001
H/CL ratio	16.75	5.75–48.80	<.0001
H/CL ratio ≥ 1.30	4.10	1.96–8.58	<.001

*G1*, grade 1; *G2*, grade 2; G3, grade 3; *H/CL*, heart-to-contralateral side ratio

**Table 3:** Multivariable analysis of patient characteristics, cardiac HMDP uptake and mortality

Variable	Hazard ratio	Confidence interval	*P* value
Total mortality (model 1)			
Age	1.06	1.05–1.08	< .0001
Female	.92	.78–1.07	.25
Bone metastasis	4.01	3.53–4.55	< .0001
Cardiac uptake (G3)	1.97	1.08–3.59	.03
Cardiovascular mortality (model 1)			
Age	1.12	1.09–1.15	< .0001
Female	.56	.36–.85	< .01
Bone metastasis	1.97	1.40–2.75	< .001
Cardiac uptake (G2)	2.19	1.21–3.94	< .01
Cardiovascular mortality (model 2)			
Age	1.12	1.09–1.15	< .0001
Female	.57	.37–.87	< .01
Bone metastasis	1.97	1.41–2.74	.0001
Cardiac uptake (G3)	4.44	1.80–10.99	.001
Cardiovascular mortality (model 3)			
Age	1.12	1.09–1.15	< .0001
Female	.55	.36–.83	< .01
Bone metastasis	1.96	1.41–2.74	< .0001
Cardiac uptake (≥G2)	2.73	1.67–4.45	< .0001

*G1*, grade 1; *G2*, grade 2; G3, grade 3; *HMDP* hydroxymethylene diphosphonate

Univariate predictors of cardiovascular mortality were suspected ATTR (≥G2), H/CL ratio, age, gender and bone metastasis (*P* < .05). High-grade cardiac uptake (grade 3) was a strong predictor of cardiovascular death compared to other variables (HR 8.7, CI 3.6-21.3, *P* < .0001). Independent predictors of cardiovascular death in multivariate analysis were suspected ATTR, age, gender and presence of bone metastasis (*P* < .05).

In patients with suspected ATTR, previous diagnosis of heart failure was associated with both elevated overall mortality (HR 2.2, CI 1.2-4.2 and *P* < .01) and cardiovascular mortality (HR 3.3, CI 1.3-8.6 and *P* = .01).

### Clinical Value of H/CL Measurements

Of the 370 patients with any degree of cardiac uptake 47 (2.4%) met the criteria for ATTR according to the H/CL threshold of ≥ 1.30. Cardiac uptake as measured by H/CL ratio was higher in patients with visually suspected ATTR (≥G2 uptake) compared to those with mild G1 cardiac uptake (1.35 ± .34 vs 1.06 ± .11, *P* < .0001). H/CL increased in each visual cardiac uptake grade G1, G2 and G3 (1.06 ± .11 vs 1.23 ± .26 vs 1.70 ± .31, *P* < .0001). As shown in Figure 2. a positive H/CL ratio (≥ 1.30) was associated with worse survival. The H/CL ratio was found to be a strong independent predictor of both overall and cardiovascular mortality in multivariable analysis (Table 4).

**Table 4:** Multivariable analysis of patient characteristics, heart-to-contralateral side (H/CL) ratio and mortality in patients with cardiac HMDP uptake (N = 370).

Variable	Hazard ratio	Confidence interval	*P* value
Total mortality (model 1)			
Age	1.07	1.05–1.10	< .0001
Female	.72	.48–1.08	.11
Bone metastasis	2.82	2.09–3.80	< .0001
H/CL ratio	3.00	1.47–6.13	< .01
Total mortality (model 2)			
Age	1.08	1.05–1.10	< .0001
Female	.72	.48–1.08	.11
Bone metastasis	2.78	2.06–3.75	< .0001
H/CL ratio (≥ 1.30)	1.68	1.07–2.64	.03
Cardiovascular mortality (model 1)			
Age	1.13	1.07–1.19	< .0001
Female	.51	.31–1.79	.51
Bone metastasis	2.19	1.10–4.36	.02
H/CL ratio	12.10	3.78–38.78	< .0001
Cardiovascular mortality (model 2)			
Age	1.14	1.08–1.20	< .0001
Female	.74	.31–1.78	.50
Bone metastasis	2.15	1.09–4.24	.03
H/CL ratio (≥ 1.30)	3.22	1.52–6.81	< .01

*G1*, grade 1; *G2*, grade 2; G3, grade 3; *H/CL*, heart-to-contralateral side ratio; *HMDP* hydroxymethylene diphosphonate

## Discussion

In this study, we evaluated the prognostic significance of incidental suspected ATTR on routine bone scintigraphy in elderly patients. Our main finding is that a high-grade cardiac uptake (grade 3) is associated with high overall mortality. In addition, cardiac uptake predicted elevated cardiovascular mortality (≥grade 2). Mild cardiac uptake (grade 1) had no impact on survival. Moreover, quantitative H/CL analysis was a strong predictor of both overall and cardiovascular mortality. To the best of our knowledge, this is the first study to describe the effect of incidental cardiac uptake on bone scintigraphy on patient mortality.

The incidence of suspected ATTR in our elderly population was 3.5% by visual analysis and 2.4% by quantitative analysis. In a previous study by Longhi et al. of more than 12 000 patients of all ages undergoing bone scintigraphy, the incidence of suspected ATTR was .4%.^6^ In their study, the majority of cardiac uptake was seen in patients being over 70 years old (93%).^6^ In another study of 800 patients cardiac uptake was seen in 2.2% patients of whom the majority were over 80 years old.^7^ Different incidences between studies might be explained by different study populations and radiotracers used. Longhi et al. used ^99m^Tc-3,3-diphosphono-1,2-propanodicarboxylic acid (DPD), Al Nahhas et al. used both ^99m^Tc-methylene diphosphonate (MDP) and HMDP while we used exclusively HMDP. Moreover, the diagnosis of cardiac uptake on scintigraphy is subjective. To increase the generalizability of our results we used the currently accepted semi-quantitative grading (Perugini grade) and a H/CL analysis.^3^,^10^-^12^ The strength of our study is the use of comprehensive national mortality statistics. Furthermore, our real-world study population is derived from three imaging centers, which serve the entire population of the area, compared to previous single-center reports.^6^,^7^

In a previous multicenter trial of 1200 patients Gillmore et al. established the good accuracy of Perugini grade classification (≥grade 2) in amyloid scintigraphy for diagnosis of cardiac ATTR.^3^ Castano et al. validated the H/CL ratio of ≥ 1.30 for diagnosis of ATTR when using a three-hour imaging protocol and demonstrated its prognostic value in patients with ATTR.^12^ Scintigraphy has been shown to detect ATTR early in its subclinical stages which raises the possibility of overdiagnosis of clinically significant ATTR in our elderly population.^1^,^8^ Nevertheless, the majority of patients undergoing bone scintigraphy have an oncologic disease and even subclinical amyloid cardiomyopathy could have implications on their surgical risk, future cardiotoxic therapies and possible thrombotic complications. In addition, atrial fibrillation combined with the pro-thrombotic state of amyloidosis might contribute to elevated mortality.^13^ Interestingly, as in previous studies, the presence of metastatic cancer was associated with increased cardiovascular mortality in our study.^14^-^16^ Oncologic patients suffering from an amyloid process, might experience additional cardiovascular complications such as endothelial dysfunction, arrythmias and myocardial infarction.^17^ However, the incidence of bone metastases was similar in the ATTR and non-ATTR groups.

A significant number of our patients (41%) had a diagnosis of heart failure at the time of bone scintigraphy which was also associated with worse prognosis. Thus, it is likely that a large portion of suspected ATTR patients on bone scintigraphy have an undiagnosed amyloid cardiomyopathy instead of a subclinical amyloid process. The poor prognosis of patients with moderate to severe cardiac uptake in our study supports further risk stratification of suspected ATTR. In contrast, mild cardiac uptake (grade 1) was not associated with elevated risk in our study and probably reflects the visualization of left ventricular blood pool activity due to delayed clearance of radiotracer in elderly individuals.^18^ Echocardiography with speckle tracking, brain natriuretic peptide measurements and selective use of cardiac MRI could be cost-effective in further risk assessment of patients with cardiac uptake.^1^,^11^,^18^ Individuals with a normal echocardiogram may have a subclinical cardiac amyloid process.^1^,^8^ Alternatively, the cardiac uptake may be due to other reasons than amyloidosis.^1^,^8^ Nonetheless, individuals with subclinical cardiac ATTR might suffer from previously undiagnosed but clinically significant non-cardiac amyloid conditions.

### Limitations

Due to the retrospective nature of our study, we do not have histological or imaging data to confirm the suspected ATTR on bone scintigraphy. In a study by Longhi et al., a subset of patients with incidental cardiac uptake underwent further biopsy assessment and all had a positive histology for ATTR.^6^ Individuals with non-cardiovascular death, frequently have coexisting cardiac morbidity but only immediate cardiovascular death was considered as an adverse cardiovascular event in our study. Other possible causes for cardiac uptake on bone scintigraphy are non-ATTR amyloidoses, acute myocardial infarction, kidney disease and hyperparathyroidism.^1^,^3^,^19^,^20^ We did not find other characteristic bone or soft tissue findings suggestive of hyperparathyroidism on suspected ATTR patients. Nevertheless, other causes of incidental cardiac uptake should be sought when clinical risk assessment of suspected incidental ATTR is performed.

In our patient population, quantitative H/CL analysis reclassified several suspected ATTR cases by visual grading only, which demonstrates the subjectivity of visual scoring and the possibility of overdiagnosis of ATTR in this low pre-test probability population. However, we cannot compare the actual accuracy of visual and quantitative analysis due to lack of information on ATTR diagnoses during follow-up. Due to our elderly population, their oncological diseases, lack of knowledge on nuclear amyloid imaging at the time of scintigraphies and considering that ATTR medications were not available in Finland further cardiological evaluations were uncommon in our study population. Furthermore, the optimal diagnostic H/CL threshold might differ in cases of incidental cardiac uptake compared to patients with clinically suspected ATTR. Our study population consists of older individuals and cannot be generalized to younger populations. We chose to include only elderly individuals into our study to increase the incidence of positive ATTR cases and considering that hereditary ATTR seen on younger individuals is rare in Finland. In addition, the majority of our study patients underwent bone imaging due to oncologic disease and thus differ from an asymptomatic normal population. Nevertheless, they represent a real-world bone scintigraphy population with incidental cardiac uptake findings.

## Conclusions

Suspected cardiac ATTR on routine bone scintigraphy is associated with elevated risk of overall and cardiovascular mortality. Further clinical evaluation of incidental cardiac uptake on bone scintigraphy is warranted.

## New Knowledge Gained

Suspected cardiac ATTR as an incidental finding on routine bone scintigraphy is associated with poor prognosis. Thus, its further clinical evaluation is warranted. Quantitative H/CL ratio offers additional risk stratification of an incidental cardiac uptake on bone scintigraphy compared to visual analysis alone.

## Electronic supplementary material

Below is the link to the electronic supplementary material.

Supplementary material 1 (WMA 10492 kb)

Supplementary material 2 (PPTX 1178 kb)

## Abbreviations

ATTR: Transthyretin amyloidosis
CI: Confidence interval
CT: Computed tomography
DPD: ^99m^Tc-3,3-diphosphono-1,2-propanodicarboxylic acid
H/CL: Heart-to-contralateral
HMDP: Hydroxymethylene diphosphonate
HR: Hazard ratio
MDP: ^99m^Tc-methylene diphosphonate
MRI: Magnetic resonance imaging
SPECT: Single-photon emission tomography
^99m^Tc: Technetium-99m
