# Supplementary material for: Prognostic significance of incidental suspected transthyretin amyloidosis on routine bone scintigraphy
Source: J Nucl Cardiol. 2020 Oct 22;29(3):1021–9. doi: 10.1007/s12350-020-02396-7 (PMC9163012; doi:10.1007/s12350-020-02396-7)
Supplement: Supplementary file 2 — Supplementary material 2 (PPTX 1178 kb) [file 12350_2020_2396_MOESM2_ESM.pptx]

## Slide 1
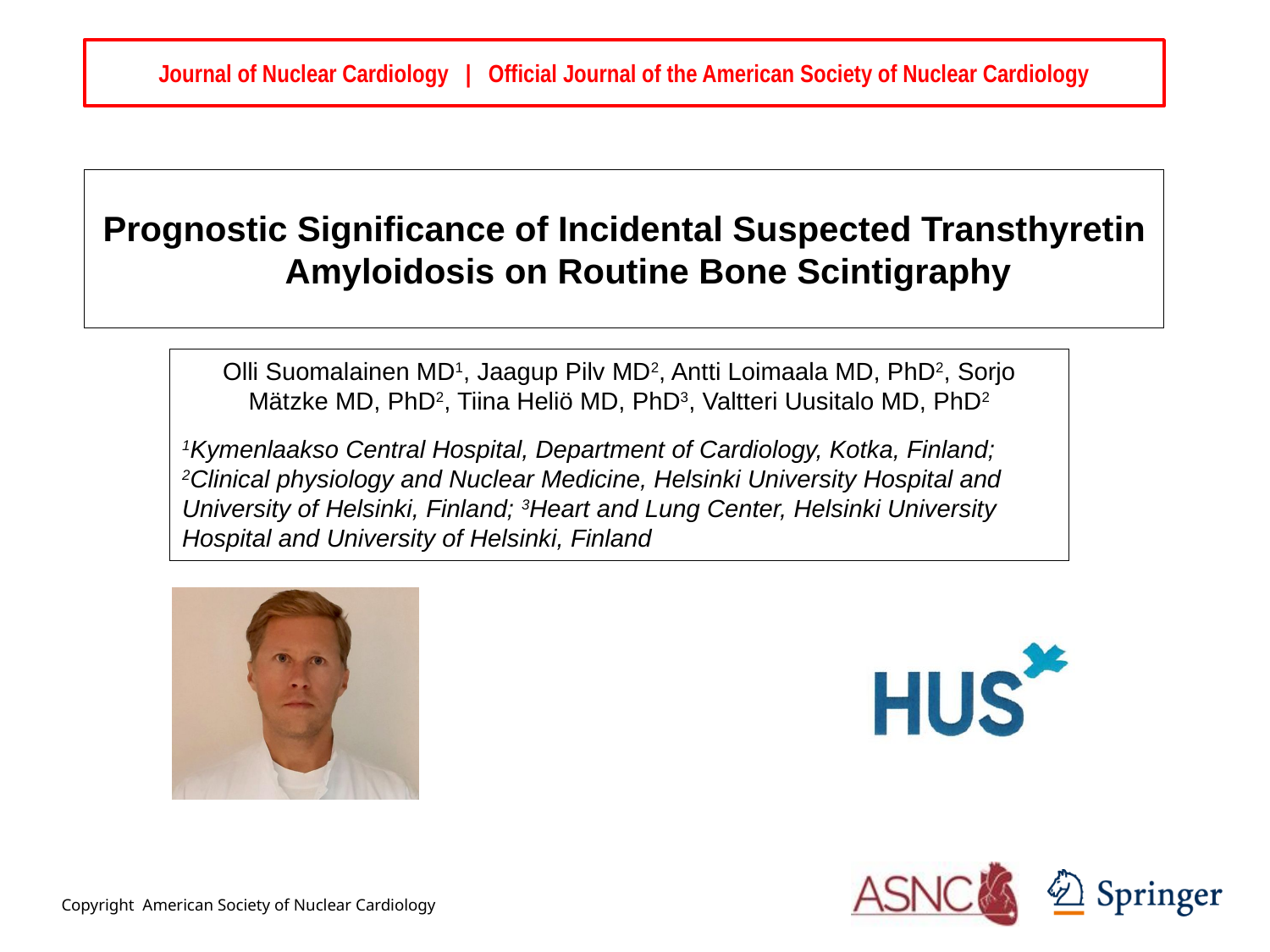

Journal of Nuclear Cardiology | Official Journal of the American Society of Nuclear Cardiology
# Prognostic Significance of Incidental Suspected Transthyretin Amyloidosis on Routine Bone Scintigraphy
Olli Suomalainen MD1, Jaagup Pilv MD2, Antti Loimaala MD, PhD2, Sorjo Mätzke MD, PhD2, Tiina Heliö MD, PhD3, Valtteri Uusitalo MD, PhD2
1Kymenlaakso Central Hospital, Department of Cardiology, Kotka, Finland; 2Clinical physiology and Nuclear Medicine, Helsinki University Hospital and University of Helsinki, Finland; 3Heart and Lung Center, Helsinki University Hospital and University of Helsinki, Finland
Copyright American Society of Nuclear Cardiology

## Slide 2
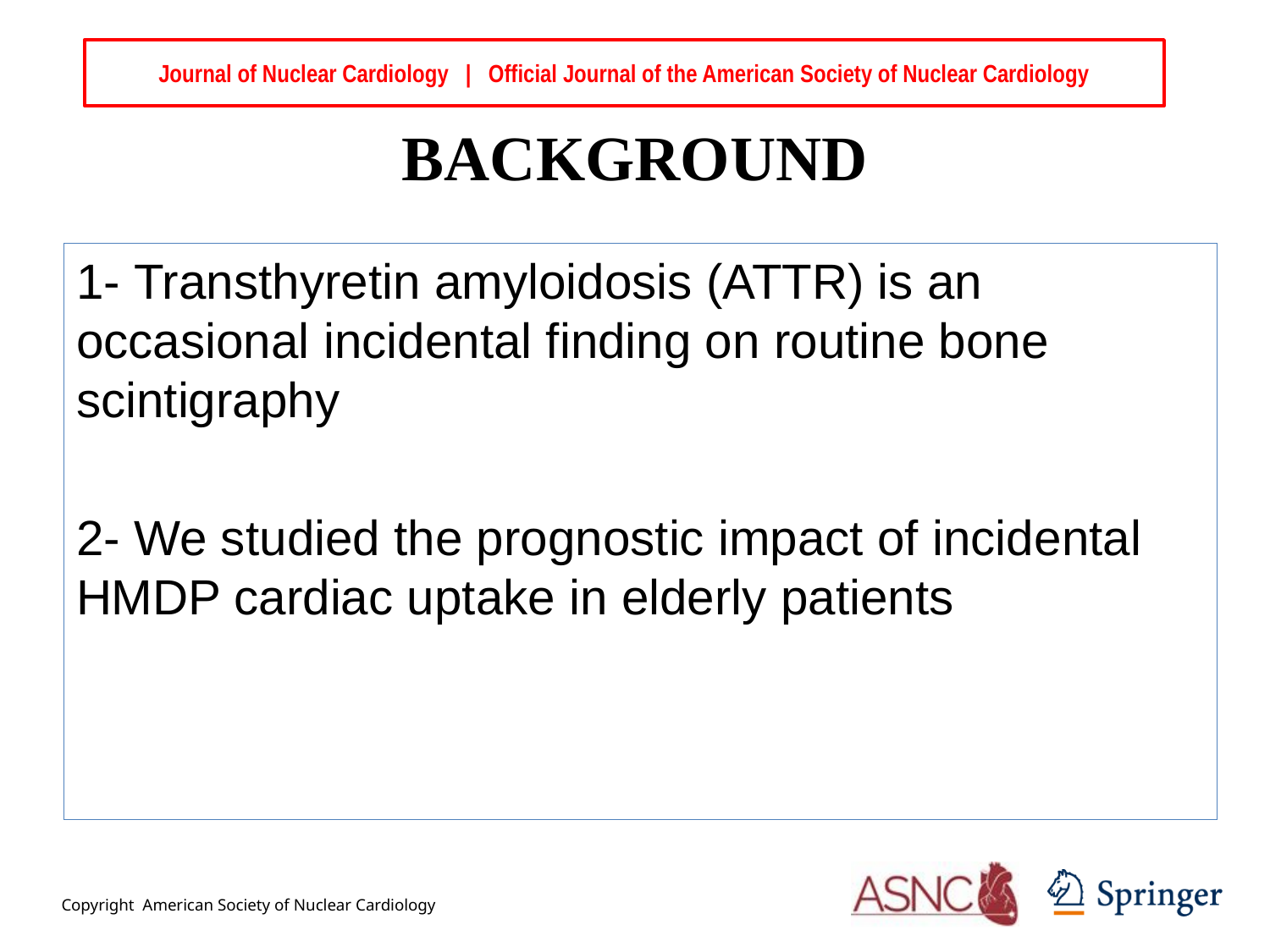

Journal of Nuclear Cardiology | Official Journal of the American Society of Nuclear Cardiology
# BACKGROUND
1- Transthyretin amyloidosis (ATTR) is an occasional incidental finding on routine bone scintigraphy
2- We studied the prognostic impact of incidental HMDP cardiac uptake in elderly patients
Copyright American Society of Nuclear Cardiology

## Slide 3
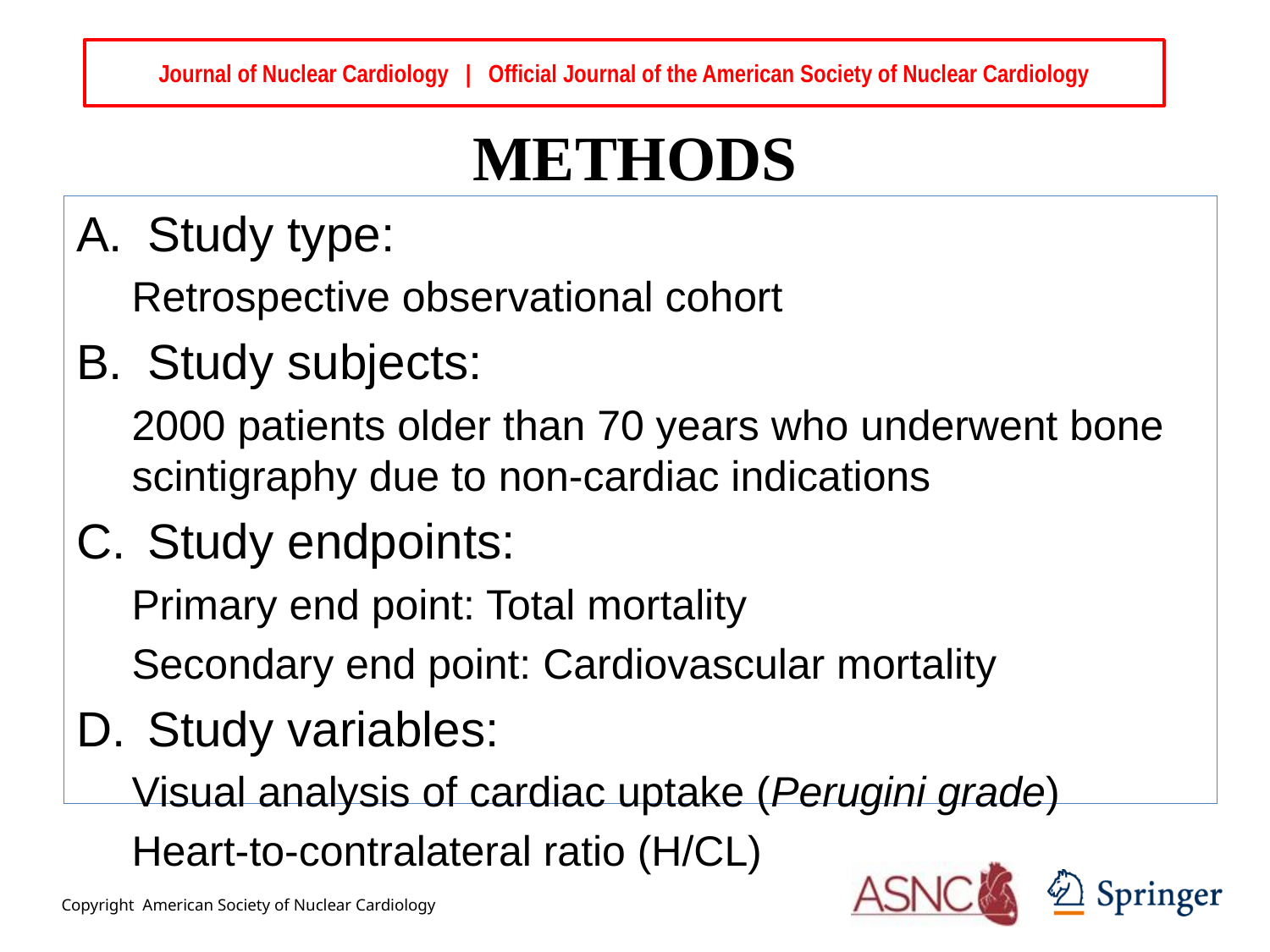

Journal of Nuclear Cardiology | Official Journal of the American Society of Nuclear Cardiology
# METHODS
Study type:
Retrospective observational cohort
Study subjects:
2000 patients older than 70 years who underwent bone scintigraphy due to non-cardiac indications
Study endpoints:
Primary end point: Total mortality
Secondary end point: Cardiovascular mortality
Study variables:
Visual analysis of cardiac uptake (Perugini grade)
Heart-to-contralateral ratio (H/CL)
Copyright American Society of Nuclear Cardiology

## Slide 4
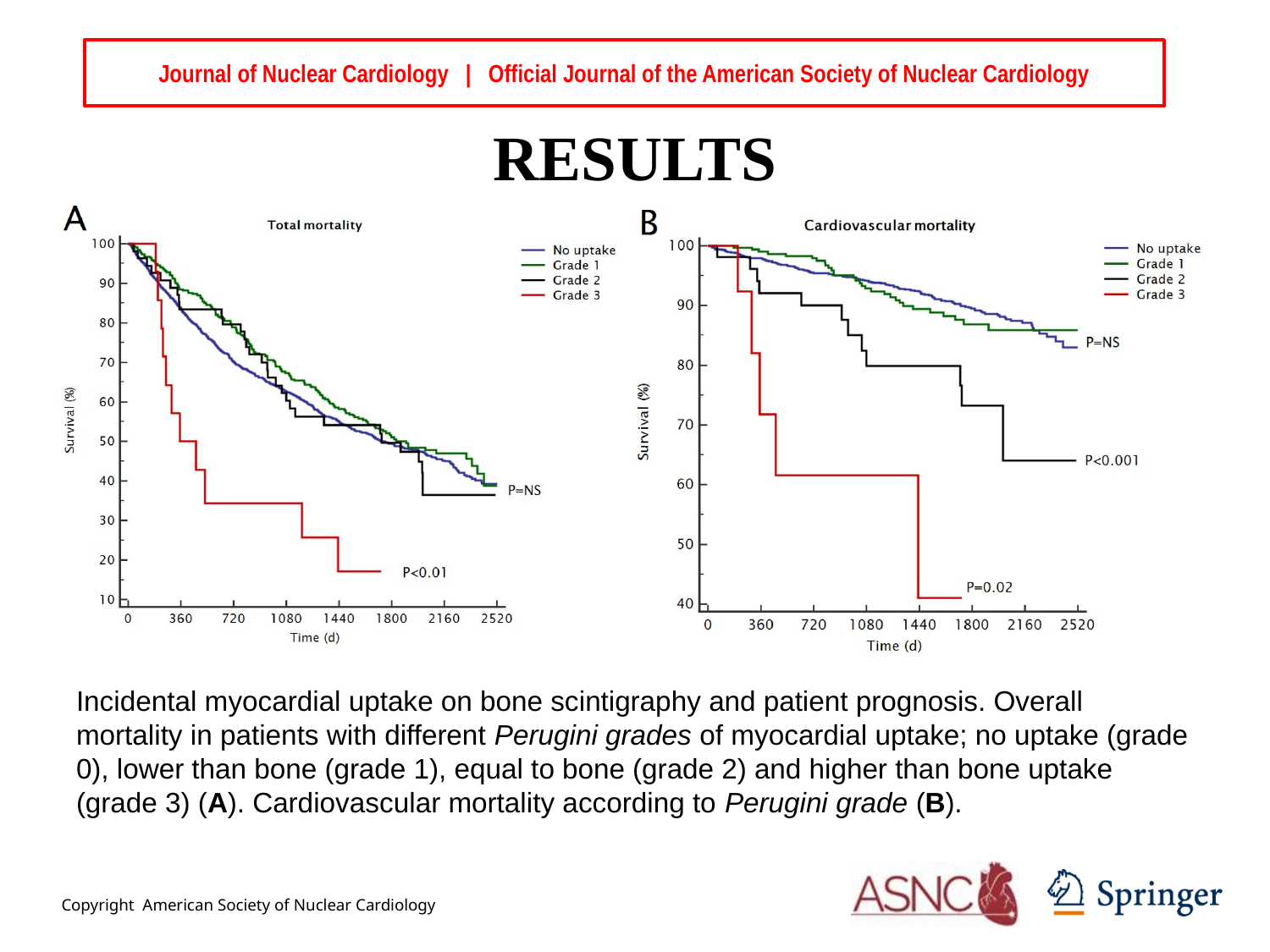

Journal of Nuclear Cardiology | Official Journal of the American Society of Nuclear Cardiology
# RESULTS
Incidental myocardial uptake on bone scintigraphy and patient prognosis. Overall mortality in patients with different Perugini grades of myocardial uptake; no uptake (grade 0), lower than bone (grade 1), equal to bone (grade 2) and higher than bone uptake (grade 3) (A). Cardiovascular mortality according to Perugini grade (B).
Copyright American Society of Nuclear Cardiology

## Slide 5
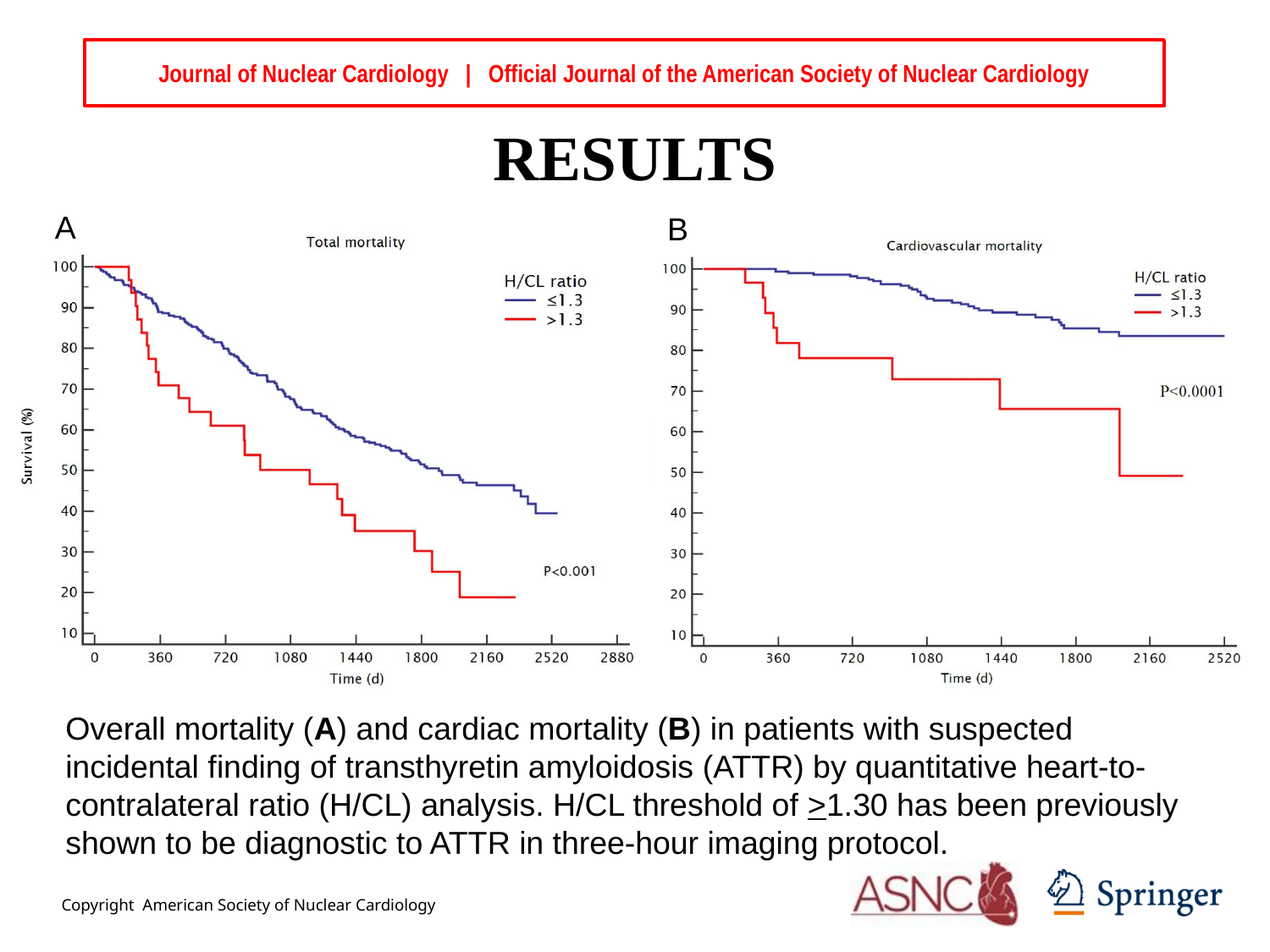

Journal of Nuclear Cardiology | Official Journal of the American Society of Nuclear Cardiology
# RESULTS
A
B
Overall mortality (A) and cardiac mortality (B) in patients with suspected incidental finding of transthyretin amyloidosis (ATTR) by quantitative heart-to-contralateral ratio (H/CL) analysis. H/CL threshold of >1.30 has been previously shown to be diagnostic to ATTR in three-hour imaging protocol.
Copyright American Society of Nuclear Cardiology

## Slide 6
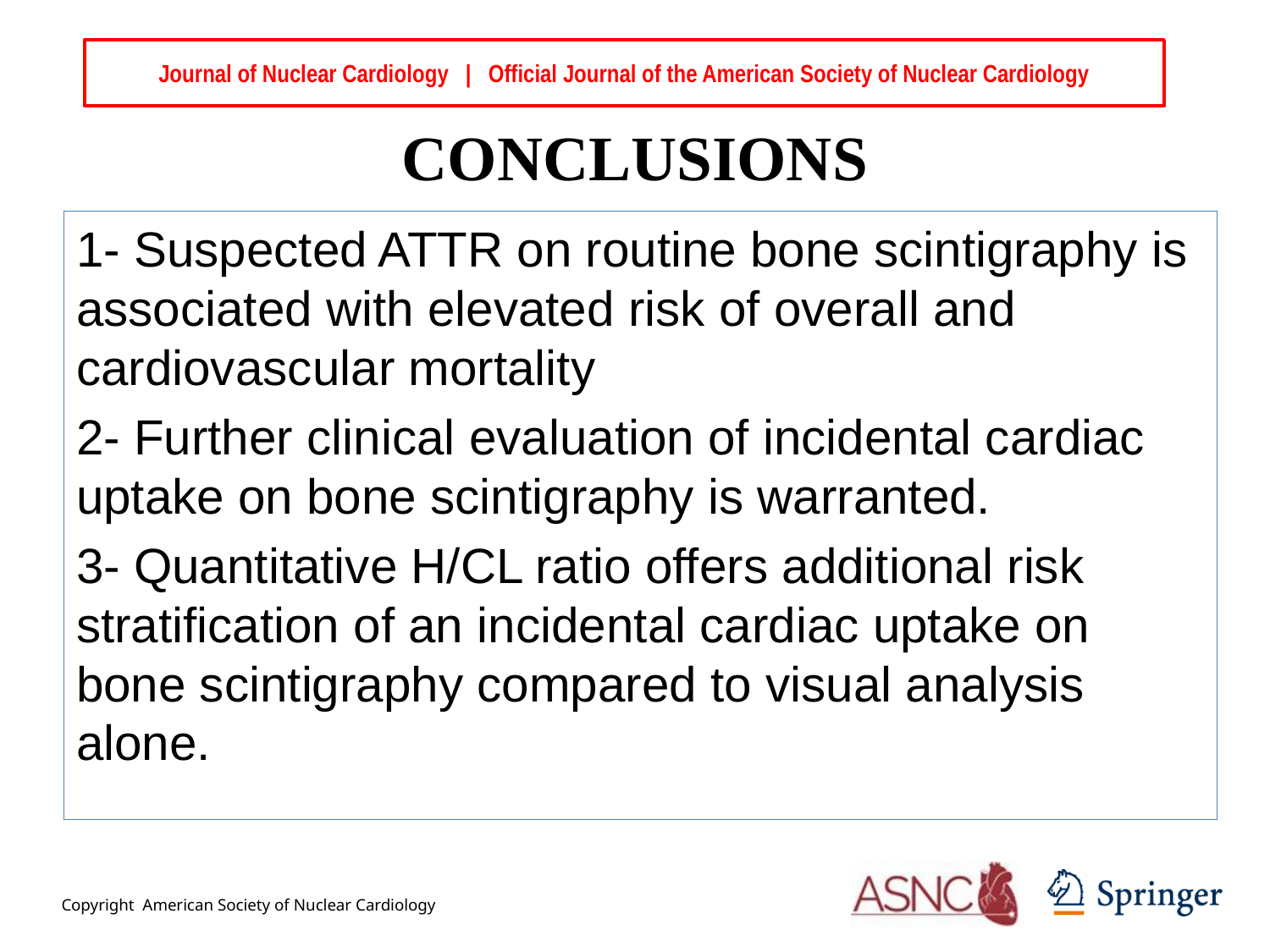

Journal of Nuclear Cardiology | Official Journal of the American Society of Nuclear Cardiology
# CONCLUSIONS
1- Suspected ATTR on routine bone scintigraphy is associated with elevated risk of overall and cardiovascular mortality
2- Further clinical evaluation of incidental cardiac uptake on bone scintigraphy is warranted.
3- Quantitative H/CL ratio offers additional risk stratification of an incidental cardiac uptake on bone scintigraphy compared to visual analysis alone.
Copyright American Society of Nuclear Cardiology
